# Mortality and other outcomes after paediatric hospital admission on the weekend compared to weekday

**DOI:** 10.1371/journal.pone.0197494

**Published:** 2018-05-21

**Authors:** Rebecca Barwise-Munro, Maryam Al-Mahtot, Steve Turner

**Affiliations:** Child Health, Royal Aberdeen Children’s Hospital, Aberdeen, United Kingdom; Dartmouth-Hitchcock Medical Center, UNITED STATES

## Abstract

Mortality is higher for adults admitted to hospital and for babies born on weekends compared to weekdays. This study compares in-hospital mortality and in children admitted to hospital on weekends and weekdays. Details for all acute medical admissions to hospitals in Scotland for children aged ≤16 years between 1^st^ January 2000 and 31^st^ December 2013 were obtained. Death was linked to day of admission. There were 570,403 acute medical admissions and 334 children died, including 83 who died after an admission on Saturday or Sunday and 251 who died following admission between Monday and Friday. The adjusted odds ratio (aOR) for a child dying after admission on a weekend compared to weekday was 1.03 [95% CI 0.80 to 1.32]. The OR for a child admitted over the weekend requiring intensive care unit (ICU) or high dependency unit (HDU) care was 1.24 [1.16 to 1.32], but the absolute number of admissions to HDU and ICU per day were similar on weekends and weekdays. We see no evidence of increased in-hospital paediatric mortality after admission on a weekend. The increased risk for admission to ITU or HDU with more serious illness over weekends is explained by fewer less serious admissions.

## Introduction

The 2015 Keogh report [1] has set the agenda for a “seven day service” in the NHS and is at least in part driven by several reports of increased mortality for adult patients admitted on weekends compared to weekdays,[2–4] and also of increased neonatal mortality for those born on weekends. [5–7] One study, which focussed on data from admissions to 29 paediatric intensive care units in the UK and Eire, concluded that mortality after emergency admission on the weekend was not associated with increased mortality.[8] Many thousands of children are admitted to hospital with acute medical conditions in the UK each day but it is not known whether mortality related to admission for this age group varies between weekday and weekend.

The primary aim of this whole population study of acute medical paediatric admissions was to describe in–hospital mortality in children presenting on weekends compared to weekdays. Our principle hypothesis was that there will be increased in-hospital mortality for children admitted at the weekend compared to weekday. To make the best use of the data available, we also compared the following characteristics of children between those admitted to hospital on a weekend and on a weekday: need for admission to an intensive care unit (ICU) or high dependency unit (HDU); duration of stay; zero day admission (i.e. admitted and discharged on the same day); readmitted to hospital; diagnosis.

## Materials and methods

### Study design

Details of each hospital admission in Scotland between 1^st^ January 2000 and 31^st^ December 2013 for individuals aged up ≤ 16 years of age were provided by the Information Services Division (ISD) of the Scottish Government in January 2015. The period of interest was chosen as part of a study into the changes in the characteristics of children admitted to hospital before and after the European Working Time Directive was introduced in 2003 (http://ec.europa.eu/social/main.jsp?catId=706&langId=en&intPageId=205) and the introduction of accident and emergency waiting times and out-of-hours service in 2004. We have recently reported trends in acute admissions from this dataset [9]. The dataset does not include children who were seen in the emergency department and discharged by paediatric medical or emergency department staff. The data do not include admissions to neonatal and postnatal wards. Elective medical admissions and elective and emergency surgical admissions were identifiable in the dataset and were excluded from the analysis to allow a focus on emergency medical paediatric admissions. Only admissions to the following significant facilities were considered: Children’s Unit, High Dependency care, Intensive Care Unit, Other (including Clinical Facilities of Standard Specialty Wards). Data were anonymised but each individual had a unique identifier which allowed linkage of admissions for an individual. A complete description of the details available are provided in S1 Table. The day, month and year (but not date) of admission were provided. Mortality was provided as an outcome in the data provided (there were no cases with “death on pass”). The study was approved by the ISD Caldicott guardian and the North of Scotland Research Ethics Committee. Data were held in the Grampian Data Safe Haven[10].

### Index for severity of presentation

The index for severity of presentation was admission to intensive care unit (ICU) or high dependency unit (HDU), and was derived by recoding ISD data. Individuals with multiple admissions to ICU and/or HDU were included.

### Other outcomes of admission

We described duration of stay and derived readmission, and these are described by NHS Improving Quality as improvement metrics[11]. The duration of stay was provided in whole days by ISD, values greater than 14 days were coded as 14 to prevent the few very long admissions skewing the mean value. Readmission within the same calendar month was derived by linking admissions within an individual with reference to the same month; a secondary variable “readmission within the same calendar month with same primary diagnosis” was created using the previously described variable linked to the primary diagnostic code; this approach addressed the potential for a second unrelated condition leading to readmission. We also derived a variable “zero day admission” which occurred when the length of stay was zero days and there was no other admission in the same calendar month.

### Diagnoses of children admitted by day of the week

Disease specific codes (Yes/No) were derived from International Classification of Diseases-10 coding provided by ISD. We first studied the five most common diagnoses in our population: vomiting and diarrhoea or viral intestinal infection (A084 or K529); upper respiratory tract infection (J069 or J00X); viral infection (B349); Asthma or Asthma other (J450, J459 or J46X); and respiratory syncitial virus (RSV) bronchiolitis or RSV not otherwise specified (J210 or J219). We then studied the following diagnoses which, in our experience, might commonly lead to ICU/HDU admission: febrile convulsion (R560); croup (J050); diabetic ketoacidosis (E101); meningococcal disease (A394 or A399 or A390D). Bacterial meningitis was included as a potentially life threatening condition (Haemophilus influenzae (G000), Streptococcus pneumoniae (G001), Streptococcus (G002), with other bacteria (G008) or unspecified bacteria (G009)).

### Analysis

Outcomes were compared between admissions on weekends and weekdays, and comparisons were made for each day with reference to Monday (the day with the highest number of admissions). The childhood population of Scotland fell from 1,046,602 in 2000 to 973,502 in 2013[12] but we did not adjust for change in population size since our focus was not on the differences between years but between weekend and weekday, and we assumed that the population remained static on weekends compared to weekdays. Odds ratios [with 95% confidence intervals] for outcomes were calculated using logistic regression adjusting for the child’s sex, age, socioeconomic status (Scottish Index of Multiple Deprivations), month and year of admission. Outcomes were death, admission to ITU/HDU and other outcomes of admission and admission with the specific conditions described previously. Having had a previous admission to hospital was included as an index of chronic condition and models where death was the outcome were run without and then with this variable. Outcomes are presented for the whole population and (except for specific conditions) also by the following age groups: 0–4.99, 5–9.99 and 10–16 years. Standard statistical software was used (SPSS version 23). Given the very large sample size, p values were not presented.

## Results

### Study subjects

There were 830,705 children admitted to hospitals in Scotland during the calendar years 2000 to 2013 and 629,248 were coded as acute admissions (the remainder were elective admissions) of which 570,403 were acute medical paediatric admissions (the remainder were acute admissions to other specialties, mostly surgical). Table 1 presents details of the children admitted. The following numbers of children were admitted on each day of the week: Monday 92,453 (16% of all admissions); Tuesday 85,728 (15%); Wednesday 83,953 (15%); Thursday 82,842 (15%); Friday 84,392 (15%); Saturday 68,005 (12%); Sunday 73,030 (13%). There were 334 deaths in hospital, 4,280 admissions to HDU or ITU, the mean and median duration of stay were 1.2 and 1 day respectively, and 38.7% of children (220,527) were discharged on the same day as admitted (“zero day admissions”). The proportion (number) readmitted within the same calendar month was 7.6% (43,557) and the proportion (number) readmitted with the same diagnosis was 4.0% (22,617).

**Table 1:** Descriptives of the characteristics of the population.

Characteristic		Descriptive
Male sex, % (number)		56% (318,173)
Median Scottish Index of Multiple Deprivations (1 = least affluent), [Interquartile range]		2 [1, 4]
Median age (years), [interquartile range]		2.3 [0.8, 6.3]
Age category	0-<5 years, % (number)	74% (422,188)
	5-<10 years, % (number)	15% (83,078)
	10–16 years, % (number)	11% (65,137)
Facility the child was admitted to (“Significant facility”)	Children’s Unit	362,388
	Intensive Care Unit	3,050
	High Dependency Unit	1,230
	Other (inc Clinical Facilities of Standard Specialty Ward)	203,704
	Other	31

### Mortality on weekend and weekday admission

There were 83 deaths after admission on a weekend and 251 deaths after admission on a weekday and the odds ratio (OR) for an admission leading to a child’s death in hospital after presenting on a weekend relative to a weekday was 1.03 [0.80, 1.32], Table 2. Of the children who died, 183 (55%) had previously had an admission and when previous admission was added to the model, the relationship between admission on weekend and mortality was unchanged (OR 1.03 [0.81, 1.33]). Seventy five deaths (22%) occurred on the same day as admission, 262 (78%) occurred within a week of admission and only one death occurred ≥14 days after admission. There was no difference in the interval between dates of admission and death for children admitted on weekends compared to weekdays. There was no difference in the proportion of deaths within different categories of cause for mortality occurring on weekends compared to weekdays, Table 3. The number of deaths per day of admission are presented in S2 Table. There was no difference in odds ratio for death after admission on a weekday across age categories (<5, 5–9.9, ≥10), S3, S4 and S5 Tables. Among other outcomes, the only difference across age categories was that children aged 5–9.9 and ≥10 years (but not <5 years) were less likely to be readmitted after an admission on a weekend day compared to a weekday, S3, S4 and S5 Tables. There was no difference in the number of deaths after admission on a weekend compared to weekday before the EWTD was introduced in 2003 and afterwards (S6 Table). Similarly there was no difference in the proportion of deaths after admission on a weekend relative to a weekday before the 2004 Emergency Department four hour waiting time limit was imposed and afterwards.

**Table 2:** Comparison of outcomes in children admitted on weekend days and weekdays. Data are presented as absolute numbers, the number of cases per day, unadjusted and adjusted odds ratios.

Outcome	Weekend day (denominator 141,035)	Weekdays (denominator 429,368)	Unadjusted odds ratio	Adjusted Odds ratio*
Total number of deaths in hospital *[number per 100*,*000 admissions]*	83 *[59]*	251 *[58]*	1.009	1.028 [0.801, 1.318]
Proportion of admissions to ITU or HDU (number) *[number per 100*,*000 admissions]*	0.9% (1,224) *[868]*	0.7% (3,056) *[712]*	1.221	1.236 [1.156, 1.322]
Proportion discharged on the same day and not readmitted (number) *[number per 100*,*000 admissions]*	37.3% (52,673) *[37*,*347]*	39.1% (167,854) *[39*,*093]*	0.929	0.930 [0.919, 0.942]
Readmitted in same month (number) *[number per 100*,*000 admissions]*	7.2% (10,181) *[7*,*219]*	7.8% (33,376) *[7*,*773]*	0.923	0.926 [0.905, 0.948]
Readmitted in same month and same primary diagnosis (number) *[number per 100*,*000 admissions]*	3.7% (5,240) *[3*,*715]*	4.0% (17,377) *[4*,*038]*	0.915	0.919 [0.890, 0.948]

*adjusted for sex, age, month and year of admission, socioeconomic status

**Table 3:** The total number of deaths after admission of a child to a hospital in Scotland and the number who died after admissions on a weekend. The groups were combined by arrest, respiratory, infection and “other” to ensure that individual cases could not be identified.

Overall category	Diagnostic groups included	ICD-10 code(s) used	Number of cases	Number of cases admitted on weekend (%)
Cardiorespiratory Arrest	Cardiac arrest	I460, I461, I469, R960	56	14 (25%)
	Sudden Infant Death	R95X		
	Respiratory arrest	R092		
Respiratory condition	Pneumonia	J13X, J14X, J151, J180, J181, J182, J189, J159, J910, B250, J690, J698	80	22 (28%)
	Bronchiolitis	J205, J210, J121, J219		
	Unspecified Lower Respiratory Tract Infection	J22X		
	Respiratory failure	J80X, J961, J969		
	Asthma	J450, J459, J46X		
Infection	Septicaemia	A400, A401, A410, A419	54	13 (24%)
	Meningococcal septicaemia	A392, A394		
	Other infection	R509, A041, B349, G049, I400, J101, J108, A86X		
	Meningitis	G002, G039, A390D		
	Vomiting and diarrhoea	A09X, A084, K529, R11X		
Other			144	34 (24%)

### Other outcomes on weekends and weekdays

#### HDU or ITU admissions

There were 1,224 admissions to HDU or ITU on a weekend (612/day) and 3,056 on a weekday (611/day), and the OR for an admissions requiring admission to HDU or ITU on a weekend compared to weekday were 1.24 [1.16, 1.32].

#### Duration of stay

The median and mean duration of stay were similar for weekend (1 and 1.2 days) and weekday (1 and 1.2 days) admissions, Table 2.

#### Zero day admissions and readmissions

When compared to admissions on weekdays, the OR for a zero day admission after presenting on a weekend were 0.93 [0.92, 0.94], the OR for being readmitted in the same month were 0.93 [0.91, 0.95] and for being readmitted with the same diagnosis were 0.92 [0.89, 0.95], Table 2.

#### Diagnoses made

The OR for an admission on a weekend being diagnosed with URTI, diarrhoea and vomiting, viral infection and RSV disease increased by between 0.3% and 7.8% when compared to weekday, S7 Table. In contrast, the OR of an admission on a weekend being for asthma, febrile convulsion, croup, diabetic ketoacidosis and meningococcal disease (i.e. which might result in an ITU or HDU admission) were increased by between 17% and 28%, S6 Table. The 400 cases of bacterial meningitis were no more likely to be admitted over a weekend or weekday.

### Outcomes by individual days of the week (with reference to Monday)

**Fig 1:**
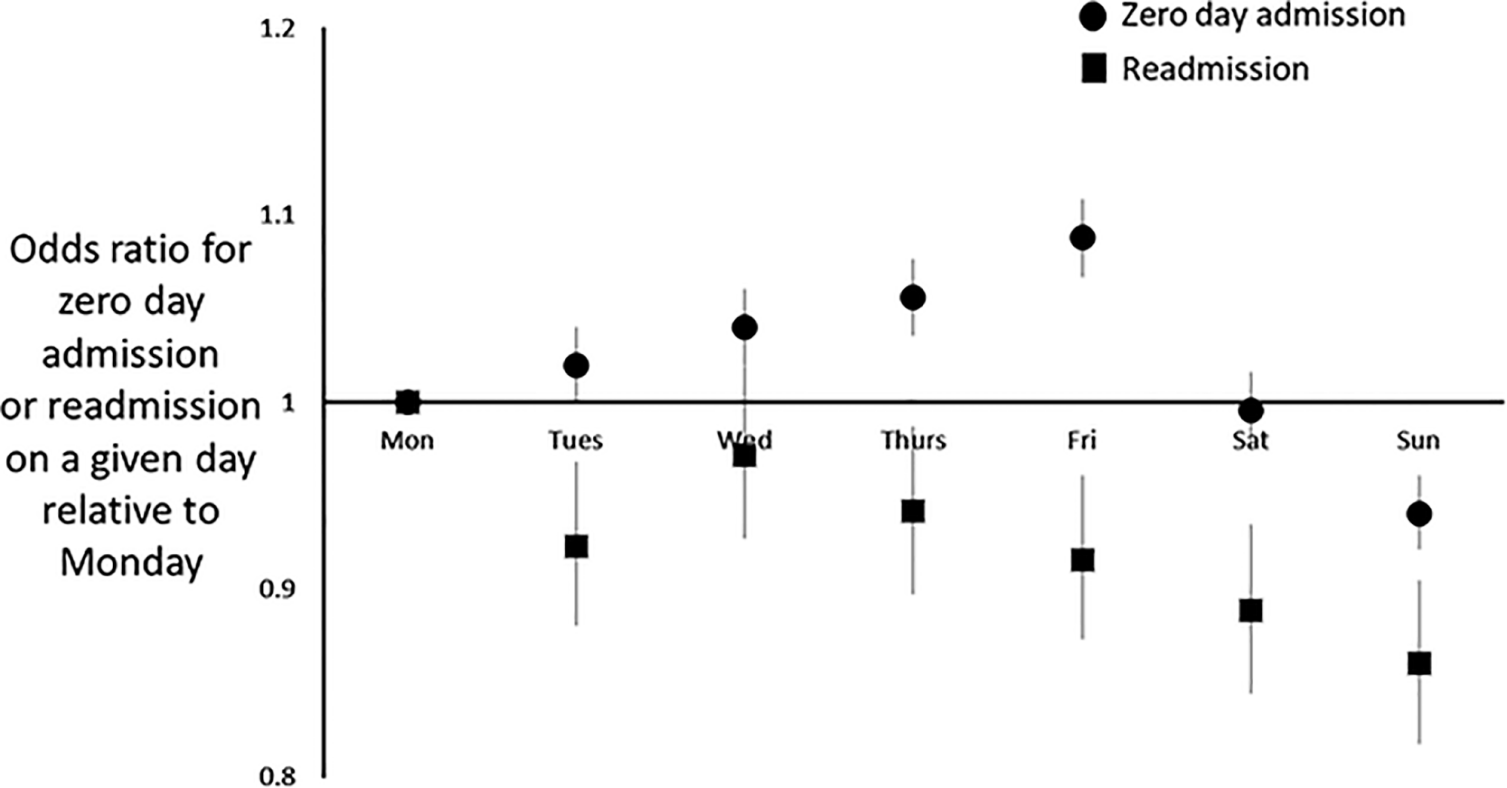
Odds ratio for zero day admission where there was no readmission within the same month (filled circles) or readmission with the same diagnosis within the same month (filled squares) on days of the week relative to Monday. The vertical lines correspond to 95% confidence intervals.

There was no increase or reduction in the OR for a child dying after admission on any day of the week. The OR for admissions to HDU/ICU were only increased on Saturday (OR 1.17 [1.05, 1.31]) and Sunday (OR 1.27 [1.14, 1.41]) and the OR for readmission with the same diagnosis were reduced on Tuesday (OR 0.92 [0.88, 0.97]), Thursday (OR 0.94 [0.90, 0.99]), Friday (OR 0.92 [0.87, 0.96]), Saturday (0.89 [0.85, 0.94]) and Sunday (OR 0.86 [0.82, 0.91]), Fig 1. The OR for zero day admission increased progressively from Monday to Friday (the OR for Friday were 1.09 [1.07, 1.11]), was not different on a Saturday and but was reduced on Sunday (OR 0.94 [0.92, 0.96]), Fig 1. Details of differences in diagnoses made across days of the week are presented in the supplement (S1 File).

## Discussion

We believe that this is the first whole-population study to compare in-hospital mortality between children admitted to hospital on a weekend or on a weekday. The main finding was that was no evidence for increased risk for mortality after presenting to hospital on weekend compared to weekend. A secondary finding was that children admitted over weekends differed to those attending on weekdays by virtue of being fewer numbers admitted and by having proportionately more severe presentations, as evidenced by ICU/HDU admission.

Our findings replicate the results from a study of paediatric ICU admissions from the UK and Eire[8] and also a single centre in the US [13]which found no evidence of increased mortality after emergency admission on weekends compared to weekdays. Our results contrast with those which have described increases in mortality for adults presenting with some conditions on weekends[2–4] and in neonates born on weekends.[5–7] The conditions presenting to acute paediatric medical receiving units are considerably different to many of those presenting to adult acute medical and special care baby units and this most likely explains the different outcomes on weekends seen in our population compared to older and younger sections of the UK population.

The Keogh report[1] rightly focusses on the patient experience and advocates timely consultant input, and paediatrics has traditionally been one of the “hands on” consultant-delivered specialties and this might partly explain the similarities for outcomes after weekend and weekday admissions that we see, eg mortality, and duration of stay. Diagnostic investigations and interventional services are rarely indicated in acute paediatric admissions and limited availability at weekends (and weekday evenings)[1] are therefore not unlikely to alter outcomes in children but may alter outcomes in adult patients. An additional potential reason for differences in mortality at weekends for children compared to neonates and adults is that mortality rates are considerably higher in the latter two groups compared to the former, and our study may have been underpowered but we see no evidence of a trend for increased mortality among more than half a million admissions so we do not believe that sample size is an issue here.

In our study, and in others, there were fewer admissions on weekends but proportionately more severe presentations and so mechanism for the reported increase in mortality in younger and older populations may partly reflect a reduction in presentations with less severe symptoms on the weekend. In our study, the risk for acute admission with chronic non-communicable conditions, especially asthma but also diabetes, fell from Monday to Friday but then peaked on Sunday and this might be explained by a lack of access to care from hospital-based specialists and also primary care at weekends. To place this in context, there were 454 more asthma admissions on Sundays compared to Fridays (equivalent to two-three cases each month in Scotland and 20–30 cases in the UK) and these are potentially avoidable admissions. Better understanding of patient, parent and healthcare practitioner decision making at weekends is required to give an insight into the different profile of patients presenting to hospitals at the weekend.

The main strength of this work is the whole (Scottish) population approach which means that the results are most likely generalisable to the rest of the UK. Although directly comparable data are not available, the average annual prevalence of admissions in Scotland and England were respectively 54 and 52 per thousand population between 2000 and 2013 and this suggests that factors leading to hospital admission are similar in both countries. A second strength is the linkage which allowed derived variables (e.g zero day admission without readmission) and protected the identity of the individuals.

We have recently described how increasing admissions to hospital are explained by very short admissions and that there was no evidence that the increase in admissions was due to increasingly severe illnesses [9]. These findings are consistent with the results presented here, where we show that the absolute number of deaths per annum has not risen between 2000 and 2013.

There are limitations to our study which reflect the nature of the routinely acquired data collected, for example zero day admissions included children who might have been in hospital for 23 hours on the same day but not a child who was admitted at 11pm and discharged three hours later, but these differences are likely to be minimised by the large population size. A similar limitation is that the way data were provided meant that readmission was only with respect to the same calendar month and not an interval of 30 days after the first presentation, this limitation will underestimate the true prevalence of readmissions. A third limitation is that the primary diagnosis may not reflect the condition of the child when referred, e.g. URTI, might have initially presented with symptoms suggestive of a “more serious” condition. Further limitations of the data available are that we were not able to compare mortality between admissions during “working hours” and out-of-hours, and we had no index of co-morbidity (although we reasoned that previous admission was a meaningful surrogate for this outcome). As with all routinely acquired data, there is the potential for some miscoding, for example the Paediatric Intensive Care Network Audit reports that there were 1714 unplanned admissions to ITU in Scotland [14] and this compares with 1374 in our study. Deaths out-of-hospital and in emergency departments are increased over Christmas Day, Boxing Day and New Year’s Day[15] and we were not able to consider this on our analysis since the date of admission was not available to us, but there are only ten public holidays in Scotland so this is unlikely to have substantially affected the outcomes reported.

Whilst these results should assure the public that the care of children in UK hospitals appears to meet the same standard on weekends and weekdays, there is no room for complacency given the increase in paediatric mortality relative to the rest of Europe [16]. The Royal College of Paediatrics and Child Health has an ambitious standard of care that all admissions are seen by a tier two trainee/middle grade within four hours and consultant within 14 hours[17] but paediatric expertise is required in the community, where the majority of child healthcare is delivered. Looking ahead, the answer to the question “how do we ensure the best healthcare for our children?” lies in joined up working across primary and secondary care supported by analysis of routinely collected data which could include medication prescribed during an admission and what contact with primary care, out-of-hours or accident and emergency services took place before and after a hospital admission.

## Supporting information

S1 Table Descriptives available for analysis. (DOCX)

S2 Table Number of children admitted in each day of the week and the number who died after being admitted on each day of the week. (DOCX)

S3 Table Comparison of outcomes in children aged under 5 years admitted on weekend days and weekdays. Data are presented as absolute numbers, the number of cases per day, unadjusted and adjusted odds ratios.
(DOCX)

S4 Table Comparison of outcomes in children aged 5-<10 years admitted on weekend days and weekdays. Data are presented as absolute numbers, the number of cases per day, unadjusted and adjusted odds ratios.
(DOCX)

S5 Table Comparison of outcomes in children aged 10 to 16 years admitted on weekend days and weekdays. Data are presented as absolute numbers, the number of cases per day, unadjusted and adjusted odds ratios.
(DOCX)

S6 Table The number of deaths and the number of hospital admissions per annum. (DOCX)

S7 Table The number of children presenting with acute medical conditions stratified by presentation at the weekend or weekday. Results are presented as absolute numbers, proportion and odds ratio and as adjusted odds ratio.
(DOCX)

S1 File Diagnoses made by individual days of the week (with reference to Monday). (DOCX)
