# Supplementary material for: Mortality and other outcomes after paediatric hospital admission on the weekend compared to weekday
Source: PLoS One. 2018 May 21;13(5):e0197494. doi: 10.1371/journal.pone.0197494 (PMC5962085; doi:10.1371/journal.pone.0197494)
Supplement: S1 Table — (DOCX) [file pone.0197494.s001.docx]

S1 Table. Descriptives available for analysis

| **Variable name** | **Description** |
| --- | --- |
| PatientID | Anon patient ID |
| SEX | Sex (1=male, 2=female) |
| AGE_IN_YEARS | Age at admission (years) |
| AGE_IN_MONTHS_ADDITIONAL | Additional months to AGE_IN_YEARS (months) |
| SIGNIFICANT_FACILITY | Significant facility (see SMR01 coding tab) |
| ADMISSION_TYPE | Type of admission (see SMR01 coding tab) |
| ADMISSION_DAY_OF_WEEK | Admission day of week (MON, TUE, etc.) |
| ADMISSION_MONTH | Admission month of year (1=Jan, 2=Feb etc.) |
| ADMISSION_YEAR | Admission year |
| DISCHARGE_DAY_OF_WEEK | Discharge day of week (MON, TUE, etc.) |
| LENGTH_OF_STAY | Length of stay (days) |
| MAIN_CONDITION | Main condition (ICD10) |
| MAIN_CONDITION_DESCRIPTION | Main condition description |
| DISCHARGE_TYPE | Discharge type (see SMR01 coding tab) |
| INPATIENT_DAYCASE_IDENTIFIER | I=Inpatient, D=Daycase |
| scsimd2012quintile | SIMD 2012 Scotland level quintile (1=most deprived; 5=least deprived) |
| hbsimd2012quintile | SIMD 2012 Health Board level quintile (1=most deprived; 5=least deprived) |
| HBTREAT | Health Board of treatment (see SMR01 coding tab) |
| SPECIALTY | AF=Paediatrics, CA=Paediatric Surgery, D8=Paediatric Dentistry |

| **Admission type:** | [ISD Scotland Data Dictionary - Admission type](http://www.ndc.scot.nhs.uk/Dictionary-A-Z/Definitions/index.asp?Search=A&ID=58&Title=Admission%20Type) |
| --- | --- |
| **Code** | **Description** |
| Emergency Admission |  |
| 30 | Emergency Admission, no additional detail added |
| 31 | Patient Injury - Self Inflicted (Injury or Poisoning) |
| 32 | Patient Injury - Road Traffic Accident (RTA) |
| 33 | Patient Injury - Home Incident (including Assault or Accidental Poisoning in the home) |
| 34 | Patient Injury - Incident at Work (including Assault or Accidental Poisoning at work) |
| 35 | Patient Injury - Other Injury (inc. Accidental Poisoning other than in the home) - not elsewhere classified |
| 36 | Patient Non-Injury (e.g. stroke, MI, Ruptured Appendix) |
| 38 | Other Emergency Admission (including emergency transfers) |
| 39 | Emergency Admission, type not known |
|  |  |
| Other Admission |  |
| 40 | Other admission types, no additional detail added |
| 42 | Maternity Admission (SMR02 only) |
| 48 | Other |
|  |  |
| Routine Admission |  |
| 10 | Routine Admission, no additional detail added |
| 11 | Routine elective (i.e. from waiting list as planned, excludes planned transfers) |
| 12 | Patient admitted on day of decision to admit, or following day, not for medical reasons, but because suitable resources are available |
| 18 | Planned transfers |
| 19 | Routine Admission, type not known |
|  |  |
| Urgent Admission |  |
| 20 | Urgent Admission, no additional detail added |
| 21 | Patient delay (for domestic, legal or other practical reasons) |
| 22 | Hospital delay (for administrative or clinical reasons e.g. arranging appropriate facilities, or test to be carried out, specialist equipment, etc.) |
|  |  |
|  |  |
| **Discharge type:** | [ISD Scotland Data Dictionary - Discharge type](http://www.ndc.scot.nhs.uk/Dictionary-A-Z/Definitions/index.asp?Search=D&ID=224&Title=Discharge%20Type) |
| **Code** | **Description** |
| Regular Discharge |  |
| 10 | Regular discharge, no additional detail added |
| 11 | Discharge from NHS inpatient/daycase care |
| 12 | Transfer within the same Provider Unit |
| 13 | Transfer to other Provider Unit |
| 14 | Patient given Extended Pass/Leave of Absence |
| 15 | Patient discharged by Mental Welfare Commission (SMR04 only) |
| 16 | Patient discharged under Community Care Order (SMR04 only) |
| 18 | Other type of regular discharge |
| 19 | Regular discharge, type not known |
|  |  |
| Irregular Discharge |  |
| 20 | Irregular Discharge, no additional detail added |
| 21 | Patient discharged himself/herself against medical advice |
| 22 | Patient discharged by relative |
| 23 | Patient absconded from detention (Record type 04 only) |
| 28 | Other type of irregular discharge |
| 29 | Irregular discharge, type not known |
|  |  |
| Death |  |
| 40 | Death, no additional detail added |
| 41 | Death - Post Mortem |
| 42 | Death - No Post Mortem |
| 43 | Death – Whilst on Pass |
|  |  |
|  |  |
| **Significant facility:** | [ISD Scotland Data Dictionary - Significant facility](http://www.ndc.scot.nhs.uk/Dictionary-A-Z/Definitions/index.asp?Search=S&ID=455&Title=Significant%20Facility) |
| **Code** | **Description** |
| 11 | Other (inc. the Clinical Facilities of Standard Specialty Ward 1K, Day Bed Unit 1J) |
| 13 | Intensive Care Unit |
| 14 | Cardiac Care Unit |
| 16 | Children's Unit |
| 17 | Accident & Emergency (A&E) Ward |
| 18 | Ward for Younger Physically Disabled |
| 19 | Spinal Unit |
| 1A | Geriatric Orthopaedic Rehabilitation Unit (GORU) |
| 1B | Rehabilitation Ward (except GORU) |
| 1C | Burns Unit |
| 1D | Geriatric Assessment Unit |
| 1E | Long Stay Unit for Care of the Elderly |
| 1F | Convalescent Unit |
| 1G | Palliative Care Unit |
| 1H | High Dependency Unit |
| 1L | Adolescent Unit |
| 1M | Transplant Unit |
| 1N | Mother and Baby Unit |
| 1P | Stroke Unit |
| 1Q | Secure Psychiatric Inpatient Facility |
| 1R | Intensive Psychiatric Care Unit (IPCU) |
| 1S | Long Stay Unit - Mental Health |
| 1T | Psychiatric Rehabilitation Unit (PRU) |
| 31 | Outpatient Department |
| 32 | Accident & Emergency Department |
| 33 | Day Hospital |
| 34 | Health Centre |
| 35 | GP Surgery Premises |
| 36 | Patient's home |
| 37 | Other Community Premises |
| 38 | Rapid Access Chest Pain Clinic |
| 39 | Ambulatory Care Hospitals |
| 40 | Acute Assessment Unit (AAU) |
|  |  |
|  |  |
| **Health Board of treatment** |  |
| **Code** | **Description** |
| S08000001 | NHS Ayrshire & Arran |
| S08000002 | NHS Borders |
| S08000003 | NHS Dumfries & Galloway |
| S08000004 | NHS Fife |
| S08000005 | NHS Forth Valley |
| S08000006 | NHS Grampian |
| S08000007 | NHS Greater Glasgow & Clyde |
| S08000008 | NHS Highland |
| S08000009 | NHS Lanarkshire |
| S08000010 | NHS Lothian |
| S08000011 | NHS Orkney |
| S08000012 | NHS Shetland |
| S08000013 | NHS Tayside |
| S08000014 | NHS Western Isles |
