# Supplementary material for: Mortality and other outcomes after paediatric hospital admission on the weekend compared to weekday
Source: PLoS One. 2018 May 21;13(5):e0197494. doi: 10.1371/journal.pone.0197494 (PMC5962085; doi:10.1371/journal.pone.0197494)
Supplement: S2 Table — (DOCX) [file pone.0197494.s002.docx]

S2 Table. Number of children admitted in each day of the week and the number who died after being admitted on each day of the week

|  | Number of children who died after admission | Number of children admitted |
| --- | --- | --- |
| Monday | 54 | 92,453 |
| Tuesday | 44 | 85,728 |
| Wednesday | 49 | 83,953 |
| Thursday | 54 | 82,842 |
| Friday | 50 | 84,392 |
| Saturday | 38 | 68,005 |
| Sunday | 45 | 73,030 |
