# Supplementary material for: Mortality and other outcomes after paediatric hospital admission on the weekend compared to weekday
Source: PLoS One. 2018 May 21;13(5):e0197494. doi: 10.1371/journal.pone.0197494 (PMC5962085; doi:10.1371/journal.pone.0197494)
Supplement: S3 Table — Data are presented as absolute numbers, the number of cases per day, unadjusted and adjusted odds ratios. (DOCX) [file pone.0197494.s003.docx]

S3 Table. Comparison of outcomes in children aged under 5 years admitted on weekend days and weekdays. Data are presented as absolute numbers, the number of cases per day, unadjusted and adjusted odds ratios.

| Outcome | Weekend day  (denominator 105,946) | Weekdays  (denominator 316,242) | Unadjusted odds ratio | Adjusted Odds ratio* |
| --- | --- | --- | --- | --- |
| Total number of deaths  *[number per 100,000 admissions]* | 52  *[49]* | 179  *[57]* | 0.867 | 0.910 [ 0.677, 1.241] |
| Proportion of admissions to ITU or HDU (number) *[number per 100,000 admissions]* | 0.9% (902)  *[851]* | 0.7% (2,219)  *[702]* | 1.215 | 1.231 [1.137, 1.331] |
| Proportion discharged on the same day and not readmitted (number)  *[number per 100,000 admissions]* | 37.9% (40,148)  *[37,895]* | 39.9% (126,259)  *[39,925]* | 0.918 | 0.916 [0.903, 0.929] |
| Readmitted in same month (number)  *[number per 100,000 admissions]* | 7.6% (8,057)  *[7,6005]* | 7.7% (24,491)  *[7,744]* | 0.980 | 0.992 [0.996, 1.018] |
| Readmitted in same month and same primary diagnosis  (number)  *[number per 100,000 admissions]* | 3.8% (4,051)  *[3,824]* | 3.8% (12,010)  *[3,797]* | 1.007 | 1.015 [0.979, 1.053] |

*adjusted for sex, age, month and year of admission, socioeconomic status
