# Supplementary material for: Mortality and other outcomes after paediatric hospital admission on the weekend compared to weekday
Source: PLoS One. 2018 May 21;13(5):e0197494. doi: 10.1371/journal.pone.0197494 (PMC5962085; doi:10.1371/journal.pone.0197494)
Supplement: S4 Table — Data are presented as absolute numbers, the number of cases per day, unadjusted and adjusted odds ratios. (DOCX) [file pone.0197494.s004.docx]

S4 Table. Comparison of outcomes in children aged 5-<10 years admitted on weekend days and weekdays. Data are presented as absolute numbers, the number of cases per day, unadjusted and adjusted odds ratios.

| Outcome | Weekend day  (denominator 20,000) | Weekdays  (denominator 63,078) | Unadjusted odds ratio | Adjusted Odds ratio* |
| --- | --- | --- | --- | --- |
| Total number of deaths  *[number per 100,000 admissions]* | 12  *[60]* | 35  *[55]* | 1.081 | 1.084 [0.562, 2.090] |
| Proportion of admissions to ITU or HDU (number) *[number per 100,000 admissions]* | 0.8% (158)  *[790]* | 0.7% (467)  *[740]* | 1.068 | 1.079 [0.898, 1.296] |
| Proportion discharged on the same day and not readmitted (number)  *[number per 100,000 admissions]* | 37.3% (7,314)  *[36,570]* | 38.4% (24,197)  *[38,360]* | 0.926 | 0.930 [0.899, 0.962] |
| Readmitted in same month (number)  *[number per 100,000 admissions]* | 6.0% (1,196)  *[5,980]* | 7.8% (4,927)  *[7,811]* | 0.751 | 0.756 [0.708, 0.807] |
| Readmitted in same month and same primary diagnosis  (number)  *[number per 100,000 admissions]* | 3.3% (659)  *[3,295]* | 4.9% (3,064)  *[4,857]* | 0.667 | 0.672 [0.617, 0.732] |

*adjusted for sex, age, month and year of admission, socioeconomic status
