# Supplementary material for: Mortality and other outcomes after paediatric hospital admission on the weekend compared to weekday
Source: PLoS One. 2018 May 21;13(5):e0197494. doi: 10.1371/journal.pone.0197494 (PMC5962085; doi:10.1371/journal.pone.0197494)
Supplement: S5 Table — Data are presented as absolute numbers, the number of cases per day, unadjusted and adjusted odds ratios. (DOCX) [file pone.0197494.s005.docx]

S5 Table. Comparison of outcomes in children aged 10 to 16 years admitted on weekend days and weekdays. Data are presented as absolute numbers, the number of cases per day, unadjusted and adjusted odds ratios.

| Outcome | Weekend day  (denominator 15,089) | Weekdays  (denominator 50,048) | Unadjusted odds ratio | Adjusted Odds ratio* |
| --- | --- | --- | --- | --- |
| Total number of deaths  *[number per 100,000 admissions]* | 19  *[126]* | 37  *[74]* | 1.704 | 1.694 [0.973, 2.949] |
| Proportion of admissions to ITU or HDU (number) *[number per 100,000 admissions]* | 1.1% (164)  *[543]* | 0.7% (370)  *[739]* | 1.475 | 1.536 [1.276, 1.849] |
| Proportion discharged on the same day and not readmitted (number)  *[number per 100,000 admissions]* | 34.5% (5,211)  *[17,270]* | 34.8% (17,398)  *[34,745]* | 0.990 | 1.002 [0.964, 1.042] |
| Readmitted in same month (number)  *[number per 100,000 admissions]* | 6.2% (928)  *[3,075]* | 7.9% (3,958)  *[7,908]* | 0.763 | 0.770 [0.715, 0.829] |
| Readmitted in same month and same primary diagnosis  (number)  *[number per 100,000 admissions]* | 3.5% (530)  *[1,756]* | 4.6% (2,303)  *[4,602]* | 0.755 | 0.757 [0.688, 0.834] |

*adjusted for sex, age, month and year of admission, socioeconomic status
