# Supplementary material for: Mortality and other outcomes after paediatric hospital admission on the weekend compared to weekday
Source: PLoS One. 2018 May 21;13(5):e0197494. doi: 10.1371/journal.pone.0197494 (PMC5962085; doi:10.1371/journal.pone.0197494)
Supplement: S6 Table — (DOCX) [file pone.0197494.s006.docx]

| Year | Number of deaths | Number of admissions |
| --- | --- | --- |
| 2000 | 30 | 33305 |
| 2001 | 27 | 34097 |
| 2002 | 23 | 34308 |
| 2003 | 24 | 35504 |
| 2004 | 21 | 37467 |
| 2005 | 29 | 40042 |
| 2006 | 27 | 43927 |
| 2007 | 22 | 44635 |
| 2008 | 21 | 44744 |
| 2009 | 28 | 44720 |
| 2010 | 17 | 41325 |
| 2011 | 29 | 43204 |
| 2012 | 23 | 46139 |
| 2013 | 13 | 46986 |

S6 table. The number of deaths and the number of hospital admissions per annum
