# Supplementary material for: Mortality and other outcomes after paediatric hospital admission on the weekend compared to weekday
Source: PLoS One. 2018 May 21;13(5):e0197494. doi: 10.1371/journal.pone.0197494 (PMC5962085; doi:10.1371/journal.pone.0197494)
Supplement: S7 Table — Results are presented as absolute numbers, proportion and odds ratio and as adjusted odds ratio. (DOCX) [file pone.0197494.s007.docx]

S7 Table. The number of children presenting with acute medical conditions stratified by presentation at the weekend or weekday. Results are presented as absolute numbers, proportion and odds ratio and as adjusted odds ratio.

| Condition (ICD-10 code) | Proportion of all weekday admissions (n=429,368) | Proportion of all weekend admissions (n=141,035) | Absolute odds ratio for being admitted on weekday compared to weekend | Adjusted Odds* ratio for being admitted on weekday compared to weekend |
| --- | --- | --- | --- | --- |
| URTI (J069 or J00X) | 7.3% (31,330) | 7.5% (10,591) | 1.032 | 1.025 [1.002, 1.049] |
| Diarrhoea and vomiting (A084 or K529) | 7.4% (31,858) | 7.9% (11,141) | 1.070 | 1.066 [1.042, 1.091] |
| Viral infection (B439) | 6.4% (27,277) | 6.8% (9,633) | 1.080 | 1.085 [1.059, 1.112] |
| RSV disease (J210 or J219) | 6.1% (26,326) | 5.9% (8,348) | 0.963 | 1.004 [0.977, 1.031] |
| Asthma (J450, J459 or J46X) | 4.5% (19,509) | 5.4% (7,603) | 1.197 | 1.203 [1.160, 1.247] |
| Febrile Convulsion (R560) | 2.3% (9780) | 2.8% (3891) | 1.217 | 1.209 [1.164, 1.256] |
| Croup (J050) | 2.5% (10,917) | 3.2% (4,464) | 1.253 | 1.249 [1.205, 1.294] |
| Bacterial meningitis (G000, G001, G002, G008 or G009) | 0.1% (305) | 0.1% (95) | 0.948 | 0.953 [0.756 1.202] |
| Diabetic ketoacidosis (E101) | 0.4% (1628) | 0.4% (589) | 1.102 | 1.171 [1.064, 1.288] |
| Meningococcal disease (A394, A399 or A390D) | 0.1% (337) | 0.1% (143) | 1.292 | 1.268 [1.040, 1.547] |

*adjusted for sex, age, month and year of admission, socioeconomic status. ICD-10=International Classification of Diseases.
