# Supplementary material for: Mortality and other outcomes after paediatric hospital admission on the weekend compared to weekday
Source: PLoS One. 2018 May 21;13(5):e0197494. doi: 10.1371/journal.pone.0197494 (PMC5962085; doi:10.1371/journal.pone.0197494)
Supplement: S1 File — (DOCX) [file pone.0197494.s008.docx]

**Diagnoses made by individual days of the week (with reference to Monday)**

The OR for an admission with asthma fell progressively from Monday to Friday (OR for Friday 0.74 [0.70, 0.79]), was no different on a Saturday but was increased on Sundays (1.08 [1.02, 1.14]), and similar, but less marked, trends were seen for RSV disease and DKA. The OR for admissions with diarrhoea and vomiting, viral infection, febrile convulsions and croup were increased only for admissions on Saturdays and Sundays with a magnitude of effect similar to that for the weekend versus weekday comparison. The OR for an admission with meningococcal disease (1.42 [1.03, 1.97]) was only increased on Sundays. The odds for an admission with URTI or meningitis did not differ over the days of the week.
